# Higher Brain Perfusion May Not Support Memory Functions in Cognitively Normal Carriers of the ApoE ε4 Allele Compared to Non-Carriers

**DOI:** 10.3389/fnagi.2016.00151

**Published:** 2016-06-24

**Authors:** Zvinka Z. Zlatar, Amanda Bischoff-Grethe, Chelsea C. Hays, Thomas T. Liu, M. J. Meloy, Robert A. Rissman, Mark W. Bondi, Christina E. Wierenga

**Affiliations:** ^1^VA San Diego Healthcare SystemSan Diego, CA, USA; ^2^Department of Psychiatry, University of California San DiegoSan Diego, CA, USA; ^3^Joint Doctoral Program in Clinical Psychology, San Diego State University/University of California San Diego (SDSU/UC)San Diego, CA, USA; ^4^Department of Radiology and Bioengineering, University of California, San DiegoSan Diego, CA, USA; ^5^Department of Neurosciences, University of California San DiegoSan Diego, CA, USA

**Keywords:** cerebral blood flow, arterial spin labeling (ASL), normal aging, brain perfusion, apolipoprotein E genotype (ApoE), verbal memory, cognition

## Abstract

Age-related changes in cerebral blood flow (CBF), which carries necessary nutrients to the brain, are associated with increased risk for mild cognitive impairment (MCI) and Alzheimer’s disease (AD). Whether the association between CBF and cognition is moderated by apolipoprotein E (ApoE) ε4 genotype, a known risk factor for AD, remains understudied, with most research focusing on exploring brain regions in which there are diagnostic group differences in CBF (i.e., cognitively normal vs. MCI vs. AD). This study measured resting CBF via arterial spin labeling (ASL) magnetic resonance imaging (MRI) and verbal memory functions using a composite score in 59 older adults with normal cognition (38 ε3; 21 ε4). Linear mixed effect models were employed to investigate if the voxel-wise relationship between verbal memory performance and resting CBF was modified by ApoE genotype. Results indicated that carriers of the ApoE ε4 allele display negative associations between verbal memory functions and CBF in medial frontal cortex, medial and lateral temporal cortex, parietal regions, insula, and the basal ganglia. Contrarily, ε3 carriers exhibited positive associations between verbal memory functions and CBF in medial frontal cortex, thalamus, insula, and basal ganglia. Findings suggest that higher CBF was associated with worse verbal memory functions in cognitively normal ε4 carriers, perhaps reflecting dysregulation within the neurovascular unit, which is no longer supportive of cognition. Results are discussed within the context of the vascular theory of AD risk.

## Introduction

Normal aging is associated with decrements in cognitive function and changes in markers of brain health, such as reductions in cerebral blood flow (CBF; Parkes et al., 2004; Lu et al., 2011). CBF, the rate of delivery of arterial blood to the capillary bed of a tissue, is a measure of brain metabolism and neural function. It can be reliably quantified using arterial spin labeling (ASL) magnetic resonance imaging (MRI), with average CBF values approximating 50 mL/100 g of tissue per minute in humans (Buxton, 2009). Compared to young adults, older individuals show CBF reductions between 18–28% (Popa-Wagner et al., 2013) at a rate of decline of 0.45% per year (Parkes et al., 2004). Age-related decreases in CBF (hypoperfusion) have been associated with cognitive decline and the development of dementia, suggesting that cerebrovascular mechanisms play an important role in brain health and the sustenance of cognitive functions (Knopman and Roberts, 2010; Popa-Wagner et al., 2013; Wierenga et al., 2014; Montagne et al., 2015).

Previous studies investigating the associations between cognition and global gray matter (GM) CBF in cognitively normal older adults report mixed results, with some showing positive associations using carotid and basilar arterial flow measurements (Rabbitt et al., 2006) and others finding negative correlations using continuous ASL MRI (Bertsch et al., 2009). For example, Heo et al. (2010) used a flow-enhanced signal intensity MRI technique to measure blood velocity and found that hippocampal blood flow was positively associated with spatial memory performance in older adults. Unfortunately, CBF measurement was limited to the hippocampus only and region of interest approaches do not allow for the detection of regional differences in specific brain regions where cognitive functions are directly correlated with CBF. A recent study by Steffener et al. (2013) employed a voxel-wise approach and found that CBF patterns related to memory scores were characterized by relative increased CBF in the cerebellum and middle orbital frontal lobe and relative decreased blood flow in the hippocampus, temporal cortex, and postcentral gyrus using ASL MRI, and that expression of the CBF pattern accounted for 44.4% of the variance in memory scores. These research findings are encouraging and warrant further investigation of the direct associations between cognition and CBF and their possible clinical implications for Alzheimer’s disease (AD) prevention.

The possible moderating effects of AD risk on the relationship between CBF and cognitive performance remain understudied. Given emerging evidence suggesting that age-related cerebrovascular dysfunction contributes to the development of AD (Kelleher and Soiza, 2013) and that CBF varies as a function of AD risk (Wierenga et al., 2012, 2013, 2014; Hays et al., 2016), a better understanding of how the association between distinct cognitive functions and regional CBF differs by AD risk status is necessary in order to identify vascular mechanisms of cognitive decline. Presence of the apolipoprotein E (ApoE) epsilon 4 (ε4) allele, which plays a role in cerebrovascular integrity and increases AD risk by 3–8 fold (Tai et al., 2016), has been shown to affect CBF in normal and pathological aging. Although results have been inconsistent, possibly due to the different methodologies employed to measure CBF and cognition, it is clear that ApoE genotype is associated with changes in CBF. For example, compared to non-carriers of the ApoE ε4 allele, ε4 carriers have shown increased CBF in the medial temporal lobes (Bangen et al., 2012), left lingual gyrus (LG), precuneus (Wierenga et al., 2013), and the right insula (Thambisetty et al., 2010) using ASL MRI (Bangen and Wierenga) and oxygen 15-labeled water positron emission tomography (PET - Thambisetty). Contrarily, areas that have shown evidence of decreased CBF in cognitively normal ε4 carriers compared to non-carriers include the left middle temporal gyrus (MTG), right inferior parietal lobe, right caudate and precentral gyrus, and right insula using ASL MRI (Kim et al., 2013). As can be seen, both increased and decreased CBF has been found in the right insula in ε4 carriers, highlighting inconsistencies in the literature, which could be explained by the use of different methodologies employed to measure CBF (i.e., ASL MRI vs. PET).

Increased CBF in the medial temporal lobe of ApoE ε4 carriers has been associated with better verbal and non-verbal memory abilities and interpreted to represent a possible compensatory mechanism for altered metabolism and/or increased demand for oxygen and glucose due to changes in neuronal activity (Bangen et al., 2012; Wierenga et al., 2012). These findings are consistent with previous research suggesting that the progression from preclinical AD to mild cognitive impairment (MCI) and later AD manifestation is characterized by a pattern of early hyperperfusion (increased CBF) needed to compensate for cerebrovascular dysregulation, followed by hypoperfusion (decreased CBF) once homeostasis can no longer be maintained due to cerebrovascular changes (Ostergaard et al., 2013; Wierenga et al., 2014). Yet, few studies have investigated whether ApoE genotype differentially moderates the direct relationship between cognitive performance and CBF (Bangen et al., 2012; Wierenga et al., 2013) and to our knowledge, no study has looked directly at this relationship using a voxel-wise approach to determine if regionally-specific associations between cognitive performance and CBF exist and are differentially affected by AD risk.

The current study used ASL MRI to investigate regions in which there was a direct relationship between CBF and verbal memory functions, and whether this association was modified by genetic risk for AD (ApoE genotype) in normal cognitive aging. We hypothesized that the direct association between verbal memory functions and CBF would be modified by ApoE genotype within regions known to subserve memory functions (prefrontal, postero-medial, temporal, and thalamus), consistent with previous reports (Grasby et al., 1993; Wierenga et al., 2012, 2013). This study differs from previous investigations since we do not seek to characterize a diagnostic group (cognitively normal vs. MCI) by ApoE (ε4 carrier vs. non-carrier) interaction on CBF, but rather investigate the moderating role of ApoE genotype in the association between CBF and verbal memory performance within a group of cognitively normal older adults, using a voxel-wise approach.

## Materials and Methods

### Participants

See Table 1 for participant demographic and cognitive characteristics. Participants were community-dwelling older adult volunteers who were enrolled in a longitudinal study of aging and/or other ongoing research studies at the University of California San Diego (UCSD) and the VA San Diego Healthcare System (VASDHS). Fifty nine participants between the ages of 65 and 88 (mean age = 72.9, SD = 6.1) were included in the current analyses, out of which 38 were non-ApoE ε4 carriers (ApoE−), while 21 were ApoE ε4 carriers (ApoE+). Normal cognitive function was determined using the empirically-derived criteria for diagnosis of MCI developed by Jak et al. (2009), whereby individuals were classified as cognitively normal if, at most, performance on one measure within one or two cognitive domains fell more than one standard deviation (SD) below age-appropriate norms. All participants underwent buccal swab DNA extraction for ApoE genotyping, a comprehensive neuropsychological evaluation to determine cognitive status and cognitive composite scores, and an MRI examination.

**Table 1:** **Participant demographic and cognitive characteristics**.

	APOE− (*N* = 38)		APOE+ (*N* = 21)	
	Mean	SD	Mean	SD	*df*	*p*
Age*	72.03	5.17	74.71	7.37	31.1	0.16
Education	16.47	2.42	16.00	2.14	57	0.428
Gender	27 female	–	15 female	–	1	0.743
DRS total score	140.92	2.44	141.24	1.9	57	0.586
Whole brain rCBF*	74.04	20.49	77.3	18.57	44.96	0.543
**Verbal memory composite**
WMS-R LM immediate recall	30.66	6.52	30.38	6.25	57	0.86
WMS-R LM delayed recall	28.76	6.89	27.14	7.81	57	0.444
CVLT-2 list 1–5 total	52.84	9.27	50.81	10.59	57	0.465
CVLT-2 SD free recall	11.58	2.96	10.33	3.12	57	0.152
CVLT-2 LD free recall	12.34	2.59	11.57	3.17	57	0.317

ApoE, Apolipoprotein E; DRS, Mattis Dementia Rating Scale; rCBF, Resting Cerebral Blood Flow; D-KEFS, Delis-Kaplan Executive Function System; CW, Color Word; WMS-R, Wechsler Memory Scale-Revised; LM, Logical Memory; CVLT-2, California Verbal Learning Test-2; SD, Short Delay; LD, Long Delay; df, degrees of freedom for independent samples T-test. *Denotes equal variances not assumed (df reported are adjusted for unequal variances).

Potential participants were excluded if they had dementia or MCI, a history of severe head injury, uncontrolled hypertension, were carriers of the ApoE ε2 allele, or had a Diagnostic and Statistical Manual of Mental Disorders-Fourth Edition Axis I diagnosis of learning disability, attention deficit disorder, mood disorder, or substance abuse. Persons with significant cerebrovascular disease, defined by Framingham Stroke Risk Profile (D’Agostino et al., 1994) 10-year probability of stroke >30%, or a history of frank stroke or coronary artery disease were excluded. In addition, participants were excluded if they had contraindications to MRI scans such as ferrous implants or a pacemaker, or if they were taking prescription psychoactive medications. No participant reported a significant level of depressive symptoms on the Geriatric Depression Scale (i.e., GDS > 10). All participants provided written informed consent prior to enrollment, and data were collected in accordance with all Ethical standards as stipulated by the Declaration of Helsinki and the UCSD and VASDHS institutional review board-approved procedures.

### Neuropsychological Assessment and Cognitive Composites

To examine the moderating role of ApoE genotype on the association between cognitive function and CBF, a verbal memory composite score was created using raw scores from tests described in Table 1. These tests were selected based on results from a principal component analysis previously reported by our group on a similar sample of older adults (Wierenga et al., 2012). Composite scores were derived by averaging the *z*-scores for each of the tests within the composite.

### Apolipoprotein E Genotyping

Genotyping for ApoE alleles was performed by the ADCS Biomarker Core at UCSD using real time PCR Restriction Fragment Length Polymorphism analysis. Genomic DNA was collected from participants using buccal swab and extracted using Qiamp DNA blood mini kit (Qiagen) followed by PCR amplification (Wierenga et al., 2012).

### MRI Acquisition

Imaging data were acquired on a GE Discovery MR750 3T whole body system with a body transmit coil and an 8-channel receive-only head coil at the University of California, San Diego’s Center for functional MRI. The structural brain sequence consisted of a high-resolution T1-weighted Fast Spoiled Gradient Recall (3D FSPGR) scan: 172 1 mm contiguous sagittal slices, field of view (FOV) = 25 cm, repetition time (TR) = 8 ms, echo time (TE) = 3.1 ms, flip angle = 12, inversion time (TI) = 600 ms, 256 × 192 matrix, Bandwidth = 31.25 kHZ, frequency direction = S-I, NEX = 1, scan time = 8 min and 13 s. Resting CBF was acquired with the Multiphase Pseudocontinuous Arterial Spin Labeling (MPPCASL) sequence, which is optimized for robust CBF quantification (Jung et al., 2010): tagging duration = 2 s, TI = 3.6 s, TR = 4.2 s, TE = minimum, reps = 64, FOV = 22 × 22 cm, 20 5 mm axial slices with a single shot spiral acquisition, collecting eight cycles where each cycle consists of eight images acquired with unique phase offsets, acquisition time (TA) = 4:46 min. A spiral scan with a long TR (4000 ms) and short TE (3.4 ms) was also acquired to obtain an estimate of the equilibrium magnetization of cerebral spinal fluid, which is used to convert the perfusion signal into calibrated CBF units (mL blood/100 g tissue/min). Finally, a minimum contrast image was acquired to adjust for transmit and receive coil inhomogeneities. Two field map scans were also acquired and used for off-line field map correction to help correct for signal bunching and dropouts in the frontal/medial temporal lobes.

### MRI Pre-Processing

Image processing was performed with Analysis of Functional NeuroImages (AFNI^1^; Cox, 1996), FMRIB Software Library (FSL, Oxford, UK), and locally created Matlab scripts. Field map correction was applied to the ASL time series prior to co-registration to the middle time point to minimize the effects of participant motion. For each participant, a mean ASL image was formed from the average difference of the control and tag images using surround subtraction to create an uncorrected perfusion time series, and slice timing delays were accounted for, making the inversion time (TI2) slice specific (Liu and Wong, 2005). This mean ASL image was then converted to absolute units of CBF (mL/100 g tissue/min) using an estimate of the equilibrium magnetization of cerebrospinal fluid (CSF) as a reference signal (Chalela et al., 2000). This procedure resulted in a calibrated perfusion value for each voxel. Skull stripping of the high-resolution T1-weighted image was performed using AFNI’s 3dSkullStrip. Scans were manually edited to remove residual non-brain material when necessary. Tissue segmentation was performed using FSL’s Automated Segmentation Tool (FAST) algorithm to define CSF, GM and white matter (WM) regions. The high-resolution T1-weighted image and partial volume segmentations were registered to ASL space, and partial volume segmentations were down-sampled to the resolution of the ASL data. To correct the CBF measures for partial volume effects and ensure that CBF values were not influenced by known decreased perfusion in WM or increased volume of CSF (Parkes et al., 2004), we used the method previously reported by Johnson et al. (2005). These calculations assume that CSF has zero CBF and that CBF in GM is 2.5× greater than that in WM. The following formula was used to compute partial volume corrected CBF signal intensities: CBFcorr = CBFuncorr/(GM + 0.4 * WM). CBFcorr and CBFuncorr are corrected and uncorrected CBF values, respectively. GM and WM are GM and WM partial volume fractions, respectively. Information from the high resolution structural image and the FSL FAST was used to determine the tissue content of each perfusion voxel. A 4.0 mm full-width half-maximum (FWHM) Gaussian filter was applied to the CBFcorr data. Voxels with negative intensities were replaced with zero (Brown et al., 2003) and GM voxels were thresholded at 0.9 probability. CBFcorr data were registered to the MNI-152 atlas using FMRIB’s Non-linear Image Registration Tool (FNIRT), part of FSL^2^ and resampled to a 3 × 3 × 3 mm resolution grid. Data were then screened for quality and outlying values deviating by more than three SDs from the mean were eliminated.

### Statistical Analyses

A voxel-wise linear mixed-effects (LME) regression model was conducted in R with voxel-wise resting CBF as the dependent variable and with independent variables: (1) verbal memory composite score; (2) ApoE status (ε4 carrier vs. ε3 carrier); and (3) the interaction term between verbal memory composite score and ApoE status. Analyses were adjusted for the effects of age. The LME yielded statistical maps displaying the brain regions for which there were significant main effects of verbal memory composite and APOE genotype on CBF and the interactive effects of ApoE status and verbal memory composite on CBF. Inferences are based on results of the ApoE status × verbal memory composite interaction term only since we aim to investigate in which brain regions there are moderating effects of ApoE status on the association between verbal memory and CBF. Significance was determined by applying cluster-size correction derived from Monte-Carlo simulations (via AFNI’s 3dClustSim) to guard against false positives on data initially thresholded at a value of *p* < 0.01 (uncorrected). Based on these simulations, it was determined that a cluster size of 19 contiguous voxels (513 mm^3^) ensured an overall *p* < 0.01. To characterize the direction of the interaction terms and obtain B values for each ApoE group, *post hoc* regression analyses were carried out using the mean CBF extracted from each significant cluster resulting from the ApoE × verbal memory composite interaction term. *Post hoc* analyses were conducted in IBM SPSS Statistics, version 22 (bootstrapped with 1000 samples) and were conducted only to characterize the significant LME interaction terms.

## Results

ApoE groups did not differ significantly on age, years of education, gender, Dementia Rating Scale (DRS) total score, whole brain resting CBF, or on any of the cognitive tests that comprised the verbal memory composite score (see Table 1).

**Figure 1:**
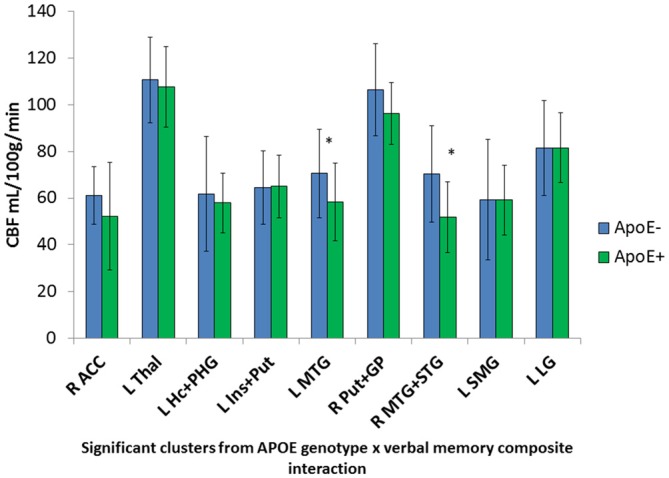
**Apolipoprotein E (ApoE) group differences in mean cerebral blood flow (CBF) in significant ApoE × verbal memory interaction clusters.** *Denotes significant ApoE group difference on CBF at *p* < 0.05. Error bars denote the standard deviation of the mean. L, Left; R, Right; ACC, Anterior cingulate cortex; Thal, Thalamus; Hc, Hippocampus; PHG, Parahippocampal gyrus; Ins, Insula; Put, Putamen; MTG, Middle temporal gyrus; GP, Globus pallidus; STG, Superior temporal gyrus; SMG, Supramarginal gyrus; LG, Lingual gyrus.

Significant interactions between ApoE genotype and verbal memory composite scores on CBF were found in nine clusters within locations consistent with distributed verbal memory processing (Grasby et al., 1993): right anterior cingulate cortex (ACC), left thalamus, left hippocampus and parahippocampal gyrus (PHG), left insula and putamen, left MTG, right putamen and globus pallidus (GP; lenticular nucleus), right middle and superior temporal gyrus (STG), left supramarginal gyrus (SMG), and the left LG. Cluster locations with coordinates and corresponding beta (B) values by ApoE genotype group are listed on Table 2. Within these nine clusters there was a trend for the ApoE+ genotype group to display overall lower CBF than the ApoE− group (Figure 1), with significant group differences in the left MTG (ApoE− mean CBF = 70.6, ApoE+ mean CBF = 58.2, *p* = 0.027) and the right middle and STG (APOE− mean CBF = 70.4, APOE+ mean CBF = 51.8, *p* = 0.011).

**Table 2:** **Interactive effects of ApoE genotype and verbal memory on cerebral blood flow (CBF)**.

	ApoE × verbal memory composite cluster locations					ApoE−		ApoE+	
Mean cerebral blood flow in:	Voxels	*X*	*Y*	*Z*	Max *F*-value	B	*p*	B	*p*
R Anterior cingulate	77	12	45	−3	20.2	11.1	0.031	−16.2	0.018
L Thalamus	60	−15	−18	0	14.3	12.5	0.014	−3.3	0.394
L Hc & PHG	49	−27	−42	−12	17.0	6.1	0.135	−13.6	0.002
L Insula & putamen	36	−30	3	12	16.4	11.1	0.015	−9.8	0.022
L MTG	34	−51	−30	−6	14.9	6.4	0.068	−18.9	0.001
R Putamen and GP	30	27	−9	−6	15.1	11.9	0.014	−5.0	0.411
R MTG and STG	28	54	−12	−12	14.6	7.3	0.142	−16.5	0.009
L SMG	25	−54	−27	18	13.5	7.3	0.055	−13.7	0.003
L Lingual gyrus	19	−6	−66	6	13.1	8.8	0.085	−10.0	0.273

*CBF, Cerebral blood flow; L, Left; R, Right; X, Y, and Z coordinates represent the peak F-value in MNI space; Hc, Hippocampus; PHG, Parahippocampal gyrus; MTG, Middle temporal gyrus; GP, Globus Pallidus; MTG, Middle temporal gyrus; STG, Superior temporal gyrus; SMG, Supramarginal gyrus*.

**Figure 2:**
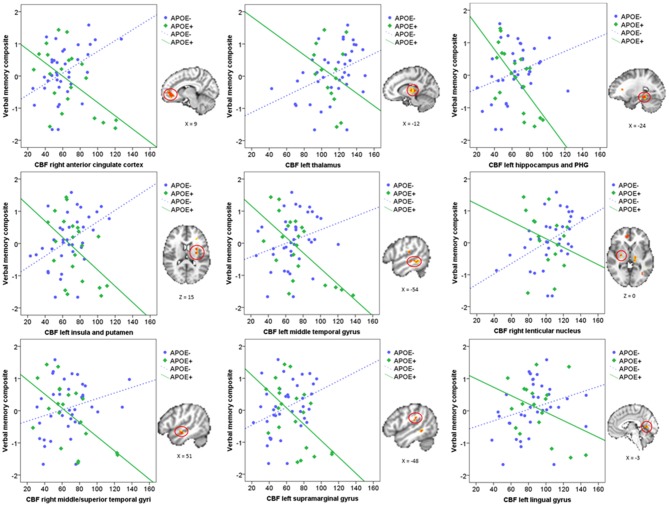
**Scatterplots characterizing the ApoE × verbal memory composite interaction on CBF in the nine significant clusters.** Green represents the ApoE+ group and blue represents the ApoE− group. Each significant cluster is depicted on the MNI152 standard brain to the right of its corresponding scatterplot. CBF, Cerebral blood flow; PHG, Parahippocampal gyrus.

Mean CBF was extracted from these nine significant clusters to characterize the direction and magnitude of the interaction effects. As can be seen in Figure 2, there is a consistent pattern of positive associations between CBF and verbal memory functions for those in the ApoE− group, whereas negative associations between CBF and verbal memory functions are observed for those in the ApoE+ group. As such, higher CBF was associated with better verbal memory function for those in the ApoE− group within the right ACC, left thalamus, left insula and putamen, and the right lenticular nucleus. Contrarily, higher CBF was associated with worse verbal memory performance for those in the ApoE+ group within the right anterior cingulate, left hippocampus and PHG, left insula and putamen, left MTG, right middle and STG, and the left SMG (see Table 2 for B-values and corresponding *p*-values). These results indicate that the interaction between verbal memory functions and ApoE genotype on CBF is characterized by positive associations for ApoE ε3 carriers and negative associations for ApoE ε4 carriers in medial frontal, medial temporal, lateral temporal and parietal regions, as well as in subcortical structures.

## Discussion

This study investigated whether ApoE genotype modified the direct relationship between voxel-wise CBF and verbal memory functions in a group of cognitively normal older adults. Our methodology differs from previous studies in two ways: (1) rather than using a region of interest approach, we aimed to investigate in which brain regions there was a significant ApoE × verbal memory performance interaction; and (2) rather than comparing CBF in cognitively normal individuals vs. those with MCI, and later examining the associations between CBF in significant regions with cognitive performance, we used verbal memory composite scores as a direct predictor of voxel-wise CBF. We believe this approach adds to the current literature by allowing us to extend previous findings onto different brain regions in which ApoE genotype may moderate the association between cognition and CBF. Results suggest that the association between CBF and verbal memory functions was modified by ApoE status in medial frontal, medial temporal, lateral temporal and parietal regions, as well as in subcortical structures. These interactions were characterized by a consistent pattern of positive associations between CBF and verbal memory functions in non-carriers of the ApoE ε4 allele, whereas for those who carry the ε4 allele, and are therefore at greater risk for developing AD, there were negative associations between verbal memory and CBF. Our findings suggest that, for those who are not at genetic risk for AD, CBF supports verbal memory functions, as expected, whereas for those carrying the ApoE ε4 allele, higher CBF is no longer supportive of verbal memory functions.

These findings are surprising given previous reports suggesting positive associations between CBF and cognition in ApoE ε4 carriers, indicative of a compensatory strategy for those at risk for AD (Bangen et al., 2012; Wierenga et al., 2012). These studies, however, investigated the association between cognitive function and CBF for a-priori regions of interest (Bangen et al., 2014) or for regions showing significant diagnostic group (cognitively normal vs. MCI) differences in CBF (Wierenga et al., 2012), rather than using a voxel-wise approach to directly examine the relationship of CBF and cognition. To our knowledge, this study represents the first to show that, for cognitively normal individuals carrying the ApoE ε4 allele, higher CBF does not seem to confer or support better cognitive performance. Typically, higher CBF should be beneficial to cognition since it provides much needed oxygen and glucose in order to support basic cognitive functions, as was the case for the ApoE ε3 carriers in this study. However, when there is damage to the cerebral vasculature, as is commonly seen in ApoE ε4 carriers (Zlokovic, 2011; Bell et al., 2012), the brain may no longer benefit from efficient use of these resources. Animal studies have shown that neurovascular coupling, the adjustment of CBF to meet the energetic demands of activated neurons, is affected in aging leading to neurovascular uncoupling. A recent study designed to pharmacologically induce neurovascular uncoupling in young mice showed that uncoupling itself was sufficient to cause cognitive deficiencies and behavioral disturbances similar to those observed in aging and pathophysiologic microvascular aging (Tarantini et al., 2015). This uncoupling within the neurovascular unit, due in part to dysregulation of production/release of endothelial and neuronal nitric oxide, may help explain why increased CBF in those at risk for AD (ApoE+ group) was associated with poorer performance compared to the ApoE− group. Moreover, studies have suggested that ApoE ε4-induced detrimental cerebrovascular changes include reduced CBF, modified neurovascular coupling, increased blood–brain barrier (BBB) leakiness, cerebral amyloid angiopathy, hemorrhages and disrupted transport of nutrients and toxins (Tai et al., 2016), all of which affect cognitive function.

The current results support a vascular-focused theory of AD risk (de la Torre, 2002; Zlokovic, 2011; Ostergaard et al., 2013), which posits that cerebrovascular damage contributes to cognitive decline and suggests that ApoE genotype modifies the association between CBF and verbal memory in normal aging. It is worth noting that these underlying cerebrovascular changes are being observed in a group of individuals without apparent cognitive impairment or clinical symptoms, further highlighting that CBF changes occur pre-clinically and may serve as an important biomarker of AD risk (Hays et al., 2016).

### Strengths and Limitations

This study was limited by a small sample size and by unbalanced ApoE groups (64.4% ApoE− vs. 35.6% ApoE+) which may affect the power to detect group differences. However, even with this small sample size we were able to find regions of significant interaction effects where the direction and magnitude of associations were consistent across brain regions. Future studies should include larger samples to replicate the current findings, as well as include individuals with MCI, who are at increased risk for AD. Furthermore, given the cross-sectional nature of this study, we cannot ascertain whether vascular dysregulation in ApoE ε4 carriers conferred worse verbal memory performance or* vice versa*. Finally, the use of ASL MRI may posit certain limitations, such as sensitivity to transit time effects, low spatial resolution, less sensitivity to WM CBF, and lack of sequence standardization across research centers, which may help to explain discrepancies in the literature. Strengths of the current study are: the inclusion of a well-characterized sample of older adults, the use of non-invasive ASL MRI to measure CBF, the availability of several cognitive test performances to characterize cognitive status, and the use of voxel-wise linear mixed effects models, which allowed us to examine the direct association between verbal memory performance and CBF within the entire brain.

## Conclusion

This study demonstrates that ApoE ε4 genotype negatively impacts or disrupts the relationship between CBF and verbal memory functions in cognitively normal older adults, whereas in non-carriers (ε3), higher CBF supported verbal memory functions. Future longitudinal studies should examine changes in CBF in cognitively normal individuals and in those with MCI over time, taking into consideration that the association between CBF and cognition may differ by ApoE status. Moreover, intervention studies that target CBF to improve cognition in aging should account for ApoE genotype, since differential changes in CBF as a function of genetic risk may impact cognitive effects.

## Author Contributions

The following authors were involved with the design (ZZZ, AB-G, MWB, CEW), acquisition (CCH, MJM, RAR), analyses (ZZZ, AB-G, CEW, CCH), and interpretation of the work (ZZZ, TTL, MWB, CEW). The manuscript was prepared by ZZZ and revised and approved by all other co-authors. All authors agree to be accountable for all aspects of the work.

## Conflict of Interest Statement

The authors declare that the research was conducted in the absence of any commercial or financial relationships that could be construed as a potential conflict of interest.

## Footnotes

^1^afni.nimh.nih.gov

^2^http://fsl.fmrib.ox.ac.uk/fsl/
